# The Effect of Visual Word Segmentation Cues in Tibetan Reading

**DOI:** 10.3390/brainsci14100964

**Published:** 2024-09-25

**Authors:** Danhui Wang, Dingyi Niu, Tianzhi Li, Xiaolei Gao

**Affiliations:** Plateau Brain Science Research Center, Tibet University, Lhasa 850000, China; wdh19980502@163.com (D.W.); lantern734004762@163.com (D.N.); litianzhi2021@163.com (T.L.)

**Keywords:** visual word segmentation cue, Tibetan reading, eye movement

## Abstract

Background/Objectives: In languages with within-word segmentation cues, the removal or replacement of these cues in a text hinders reading and lexical recognition, and adversely affects saccade target selection during reading. However, the outcome of artificially introducing visual word segmentation cues into a language that lacks them is unknown. Tibetan exemplifies a language that does not provide visual cues for word segmentation, relying solely on visual cues for morpheme segmentation. Moreover, previous studies have not examined word segmentation in the Tibetan language. Therefore, this study investigated the effects of artificially incorporated visual word segmentation cues and basic units of information processing in Tibetan reading. Methods: We used eye-tracking technology and conducted two experiments with Tibetan sentences that artificially incorporated interword spaces and color alternation markings as visual segmentation cues. Conclusions: The results indicated that interword spaces facilitate reading and lexical recognition and aid in saccade target selection during reading. Color alternation markings facilitate reading and vocabulary recognition but do not affect saccade selection. Words are more likely to be the basic units of information processing and exhibit greater psychological reality than morphemes. These findings shed light on the nature and rules of Tibetan reading and provide fundamental data to improve eye movement control models for reading alphabetic writing systems. Furthermore, our results may offer practical guidance and a scientific basis for improving the efficiency of reading, information processing, and word segmentation in Tibetan reading.

## 1. Introduction

The word is the smallest unit of language that can be utilized independently [1]. To comprehend sentences and larger textual structures, such as paragraphs and chapters, words must first be processed and recognized [2]. Words in texts must be segmented to achieve word processing and recognition; this process is referred to as word segmentation [3,4]. Word segmentation plays a significant role in information and cognitive processing during reading [5,6], and visual word segmentation cues are crucial in the word segmentation process [6,7].

Previous studies have shown that in languages with within-word segmentation cues, removing or substituting the original visual segmentation cues in a text leads to an increase in lexical recognition time and a decrease in reading speed for readers, and adversely affects saccade target selection during reading, and causes the initial fixation position on words to shift from the preferred viewing location to the beginning of the word [7,8,9,10,11,12,13,14,15]. Some studies have examined the effects of inserting artificial visual segmentation cues, such as spaces, into languages that do not have within word segmentation cues. In Thai texts, the practice of inserting spaces between words has been found to facilitate lexical recognition, though it has a detrimental effect on reading speed. It is noteworthy that this spacing does not influence saccade target selection during reading [16]. Conversely, in Japanese texts that incorporate a mix of hiragana, katakana, and kanji scripts, the addition of spaces between words does not have any significant impact on reading speed, lexical recognition, or saccade target selection [17]. When it comes to Chinese texts, the insertion of spaces between words does not interfere with the reading process [18,19]. However, it does have a positive effect on lexical recognition and aids in saccade target selection [19,20]. In summary, the effects of inserting spaces in text are inconsistent across different languages. This inconsistency may be due to inherent differences between languages.

Furthermore, some researchers believe that inserting spaces in languages that lack visual word segmentation cues changes the original spatial distribution of sentences, extends the physical length of the sentences, and reduces the processing efficiency of the reader’s parafoveal region. The addition of spaces causes the words near the fixated word to be farther from the fixation, which may result in the words near the fixation falling into a visual area with lower visual acuity, thereby reducing the benefits of the parafoveal preview [21,22,23].

To eliminate the influence of the irrelevant factor on experimental results mentioned above, researchers in recent years have adopted color alternation markings as a visual word segmentation cue [21,22,23,24,25,26,27]. Unlike spaces, color alternation markings do not extend the physical length of the entire sentence or change its spatial distribution and maintain parafoveal processing efficiency of readers [21,22,23]. Researchers have examined the effect of color alternation markings as a visual word segmentation cue in reading. However, the results of studies using eye-tracking technology with university students who are native Chinese speakers to examine the effect of color alternation markings as a visual word segmentation cue in reading are inconsistent. Some studies have shown that color alternation markings between words have no effect on reading but aid in saccade target selection and facilitated lexical recognition under certain conditions [23]. Other studies have indicated that color alternation markings between words hinder reading, do not facilitate lexical recognition, and do not affect saccade target selection [19]. Among languages that inherently lack visual word segmentation cues, studies have examined the effect of color alternation markings as a visual word segmentation cue in reading, lexical recognition, and saccade target selection only in Chinese. Therefore, whether the results can be generalized to other languages that inherently lack visual word segmentation cues remains to be verified.

Tibetan is a phonetic script belonging to the Sino-Tibetan language family. The Tibetan writing system consists of thirty basic consonant letters and four basic vowel signs, which can be combined in various ways to represent different morphemes. In the Tibetan language, the morpheme “བོད” (meaning “Tibet” or “Tibetan”) consists of the consonant “བ” combined with the vowel sign “ོ” to form “བོ”, and the consonant “ད”. Tibetan text inherently possesses a unique visual morpheme segmentation cue in the form of tshegs (“་”). For example, in the Tibetan language, the word “བོད་ཡིག” (meaning “Tibetan script”) consists of “བོད”, which means “Tibet” or “Tibetan”, and “ཡིག”, which means “script” or “writing”. The tsheg between these two morphemes acts like a delimiter, indicating that they are separate morphemes, even though together they form a single unit of meaning. Although there are morpheme segmentation cues in the Tibetan language, there are no word segmentation cues in Tibetan [28,29,30]. Furthermore, the impact of artificially inserting visual word segmentation cues on the reading of Tibetan texts is currently unknown.

Examining the effect of artificially inserting visual word segmentation cues in Tibetan reading can help address the theoretical issue of the basic information processing unit in Tibetan reading. In languages with inherent spaces between words, such as English, researchers generally consider the word a basic information processing unit [31,32,33,34,35]. Conversely, in other languages, such as Tibetan and Chinese, there are no visual word segmentation cues aside from punctuation marks denoting semantic units and pauses [36,37,38,39,40,41,42]. This lack of visual word segmentation cues has created controversy over the basic information processing unit in the reading process of these languages. Therefore, the basic information processing units in Tibetan reading remain unknown.

Wang et al. (2023) found that removing tshegs interferes with Tibetan reading [43]. This indicates that native Tibetan speakers rely on visual morpheme segmentation cues provided by tshegs between morphemes to read words, suggesting that morphemes might be the basic information processing unit in Tibetan reading. Furthermore, since the discovery of the effect of word frequency on Chinese reading provides strong evidence that words are the basic information processing units in Chinese reading [41], and Gao et al. (2020) and Li et al. (2022) identified a word frequency effect in Tibetan reading, we hypothesized that words may also be the basic information processing unit in Tibetan reading [44,45]. However, previous studies have not determined whether words or morphemes are the basic information processing unit in Tibetan reading.

Therefore, this study investigated the effect of visual word segmentation cues in Tibetan reading to explore whether these cues facilitate reading and lexical recognition, and aid in saccade target selection. Moreover, this study aims to identify the basic information processing unit in Tibetan reading.

Based on previous research that has investigated languages that do not have within-word segmentation cues [16,17,18,19,20], this study proposes the following hypotheses:

(1) Interword spaces have no effect on Tibetan reading but facilitate lexical recognition and aid in saccade target selection. The existence of spaces between words does not affect reading metrics, including average fixation duration, average saccade amplitude, number of fixations, sentence reading time, number of forward saccades, and number of regressions. Conversely, interword spaces positively impact lexical recognition metrics, such as first fixation duration, gaze duration, total fixation duration, number of first-pass fixations, total number of fixations, and refixation probability. Additionally, interword spaces enhance the metric of saccade target selection, specifically the average initial fixation position.
(2) Color alternation markings have no effect on Tibetan reading but facilitate lexical recognition and aid in saccade target selection. The existence of spaces between words does not influence reading metrics, including average fixation duration, average saccade amplitude, number of fixations, sentence reading time, number of forward saccades, and number of regressions. Conversely, interword spaces positively impact lexical recognition metrics, such as first fixation duration, gaze duration, total fixation duration, number of first-pass fixations, total number of fixations, and refixation probability. Additionally, the presence of interword spaces enhances the measure of saccade target selection, specifically the average initial fixation position.
(3) Words are more likely to be the basic information processing unit in Tibetan reading than morphemes, and words possess greater psychological reality. In other words, readers demonstrate superior performance in the areas of reading, lexical recognition, and saccade target selection when exposed to the word spacing condition and the word boundary color alternation marking condition, in contrast to their performance under the morpheme spacing condition and morpheme boundary color alternation marking condition.

## 2. Experiment 1

This experiment employed eye-tracking technology and used Tibetan university students as participants. Four visual segmentation cues were set up (normal sentence, word spacing, morpheme spacing, and non-word spacing) to investigate the following two questions: (1) whether interword spaces can facilitate Tibetan reading and lexical recognition, and whether they are helpful for saccade target selection during the reading process; (2) whether the basic information processing unit in Tibetan reading is the morpheme or the word.

### 2.1. Materials and Methods

#### 2.1.1. Participants

To ensure high statistical power, the sample size was calculated using G*Power 3.1.9.7 based on the existing literature [46,47]. The results determined that 36 participants were required for a statistical power of 0.95. In order to maintain an adequate dataset in light of possible participant withdrawals and incomplete responses, we enlisted 72 university students from Tibet (36 men and 36 women; average age: 20.43 ± 1.47 years). All participants were native Tibetan speakers with normal intelligence, normal or corrected vision, and no physical or mental illnesses. All participants were right-handed. This experiment was approved by the ethics committee of Tibet University. All participants provided written informed consent prior to participation. Participation was voluntary. The participants were compensated after completion of the experiment.

#### 2.1.2. Experimental Design

**Figure 1:**
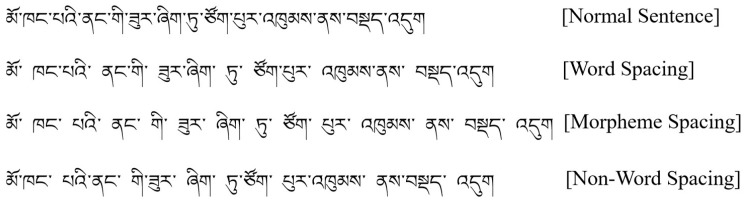
A sample Tibetan sentence displayed with different visual conditions. Translation: She is squatting in the corner of the room. The corresponding English meaning of each word in this Tibetan sentence is as follows: མོ་—she; ཁང་པའི་—the room; ནང་གི་—of; ཟུར་ཞིག་—the corner; ཏུ་—in. “ཙོག་པུར་” and “འཁུམས་ནས་” describe a squatting posture, modifying the word “བསྡད་འདུག”, which means “is squatting”. In Tibetan, prepositions often follow their corresponding nouns (“ནང་གི་—of” is placed after “ཁང་པའི་—the room”), and verbs are typically placed at the end of the sentence (“བསྡད་འདུག—is squatting” is placed at the end of the sentence).

The experiment employed a single-factor within-subjects design with four visual segmentation cues: normal sentence, word spacing, morpheme spacing, and non-word spacing. Normal sentence refers to a sentence that has not been subjected to any visual segmentation processing, indicating that there are no spaces between the words. Word spacing refers to the practice of adding spaces between words to define the boundaries between them. Morpheme spacing involves inserting spaces between each morpheme within a word, which would separate each morpheme. Non-word spacing involves inserting spaces between the morphemes in a sentence, with the spaces on both sides of a non-word unit, which is a unit larger than a morpheme but not a word. Examples of these four conditions are illustrated in Figure 1.

#### 2.1.3. Experimental Materials

Following previous studies [43], Tibetan university textbooks and extracurricular reading materials at an equivalent level were adapted to form 120 Tibetan declarative sentences. All sentences were free from syntactic and semantic ambiguities, with sentence lengths controlled at 25–30 horizontal alphabetic space lengths. After sentence compilation, 15 Tibetan university students were asked to rate the difficulty and fluency of the sentences on a five-point Likert scale (1 = very easy/very fluent; 5 = very difficult/not fluent). The results were *M* = 1.22 (standard deviation [*SD*] = 0.14) for difficulty, and *M* = 1.11 (*SD* = 0.10) for fluency. In addition, based on the standards of the Modern Tibetan Frequency Dictionary [30], the aforementioned sentences were segmented into words. A total of 15 Tibetan university students, who did not participate in the evaluation described above, were asked to rate the consistency of word segmentation. Consistency was 90.73%. After further consideration to balance the sentence length, 92 of 120 sentences were selected (sentence length: *M* = 27.83, *SD* = 1.59), of which 80 were used as experimental sentences and 12 as practice sentences. The participants who rated sentence difficulty, fluency, and word segmentation consistency did not participate in the subsequent experiments.

In the experiment, sentences were presented in four conditions: normal sentence, word spacing, morpheme spacing, and non-word spacing. Spaces equivalent to the width of a horizontal alphabetic space were inserted between the words or morphemes of the sentences. The method of inserting spaces follows the standard procedures established in previous similar studies [18,19,48,49]. Based on the four conditions mentioned above, the experiment was divided into four blocks, each containing 80 sentences, with 20 sentences for each condition. The experimental conditions were rotated according to the Latin square sequence, with each participant reading one block. Before the formal experiment, twelve practice sentences were presented, with three sentences for each experimental condition. Moreover, to ensure that the participants read attentively and understood correctly, 32 reading comprehension questions based on the content of the formal experimental materials were used. The participants were required to make a “yes” or “no” judgment, with the correct response being “yes” for 16 questions and “no” for 16 questions.

#### 2.1.4. Experimental Apparatus

The EyeLink1000Plus eye-tracking system produced by SR Research Ltd. of 35 Beaufort Drive, Ottawa, ON, Canada with a sampling rate of 1000 Hz was used. Data were collected using the default settings for cognitive research (saccade velocity threshold: 30°/s; saccade acceleration threshold: 8000°/s^2^; saccade motion threshold: 0.1°) [43,50]. A 24.5-inch DELL monitor with a screen refresh rate of 240 Hz and a resolution of 1920 × 1080 pixels, manufactured by Wistron Corporation in Zhongshan, China, was used. The distance between the participants’ eyes and monitor screen was about 65 cm, with each horizontal alphabet morpheme subtending at a visual angle of approximately 0.6°. Based on previous studies [51], the material font was Microsoft Himalaya, size 36, with one sentence displayed on each screen. Reading comprehension questions were randomly presented after presenting several experimental sentences to ensure that the participants read them carefully.

#### 2.1.5. Experimental Procedure

The experiment was conducted individually for each participant. After entering the laboratory, the participants familiarized themselves with the laboratory environment. They were then asked to sit at a designated experimental station and silently read the instructions displayed on the monitor. After the participants indicated that they had finished reading, the experimenter briefly summarized the instructions to ensure that they correctly understood the experimental process and responses required during the experimental process. The experimenter explained that once the experiment began, Tibetan sentences would be presented one at a time on the monitor screen, and participants must read each sentence carefully at their normal reading speed, ensuring that they understood the meaning of the sentences. Furthermore, the participants were informed that after several sentences were presented, a reading comprehension question regarding the previous sentence would appear. The participants were told the location of the page-turning key and true-or-false response keys. They were instructed to press the page-turning key while focusing on the fixation point on the left side of the screen center to start reading the next sentence.

Calibration was performed to ensure that the eye-tracking system could accurately record the participants’ eye movement trajectories. Three-point calibration was used, and calibration errors were controlled to be below 0.25 [43].

Subsequently, the formal experiment began. All tasks required approximately 20 min to complete. If participants experienced eye fatigue during the experiment, they were told to inform the experimenter and request a short break.

**Figure 2:**
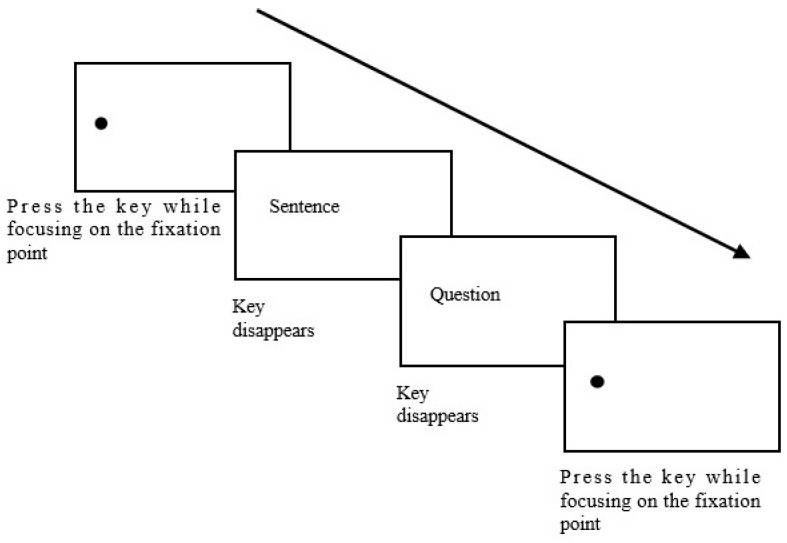
The flowchart of the experimental procedure.

If the participant could not focus on the calibration point, recalibration was performed. The trial process is illustrated in Figure 2.

#### 2.1.6. Experimental Indicators

Drawing on previous literature [18,19,52], we selected the average fixation duration, average saccade amplitude, number of fixations, sentence reading time, number of forward saccades, and number of regressions as global analysis indicators. The average fixation duration refers to the mean duration of all fixations in a sentence. The average saccade amplitude is the mean distance of all saccades from one fixation point to the next within a given sentence. The number of fixations, which reflects the cognitive processing load of the reading material, is the number of all fixation points in a sentence, with more fixation points suggesting a higher cognitive load [53]. Sentence reading time is the duration of reading a sentence from the beginning to the end and is sensitive to slower or longer cognitive processing [53]. The number of forward saccades and regressions refers to the number of forward and backward saccades made during reading.

In line with previous literature [19,23,24], we selected first fixation duration, gaze duration, total fixation duration, number of first-pass fixations, total number of fixations, refixation probability, and average initial fixation position as local analysis indicators. First fixation duration is the duration of the first fixation on an area of interest. Gaze duration is the sum of all fixation times on an area of interest, from the moment the first fixation enters until it leaves the area. Total fixation duration includes all fixation times on an area of interest, including any regressions. The number of first-pass fixations is the number of fixations on an area of interest during the first pass before leaving the area. The total number of fixations is the overall count of fixations made on an area of interest throughout the entire reading session, summing first-pass and any subsequent fixations. Refixation probability is the likelihood that a word or area of interest will be fixated upon again after the first pass. The average initial fixation position is the average position within an area of interest where the first fixation lands. First fixation duration, gaze duration, and number of first-pass fixations are indicators of early lexical processing efficiency. Total fixation duration and total number of fixations are indicators of late lexical processing efficiency [53].

### 2.2. Data Analysis

The average answer accuracy was 90.15%, indicating that the participants carefully read and understood the sentences. Following the existing studies [18,52,54], data were excluded based on the following four criteria: (1) premature or incorrect key presses that interrupted sentence presentation, (2) invalid data due to loss of tracking, (3) fixation durations of less than 80 ms or greater than 1200 ms, and (4) data points more than three SDs from the mean. Consequently, 1.98% of the total data in the global analysis and 4.15% of the total data in the local analysis were excluded due to being invalid.

Data analysis was conducted in the R programming environment (R Core Team, 2021, version 4.1.0) using the lme4 package (version 1.1-26) to build linear mixed models (LMMs) and generalized LMMs (GLMMs) [55,56]. Before running the LMMs, log transformations were applied to indicators, including the average fixation duration, average saccade amplitude, number of fixations, number of forward saccades, number of regressions, first fixation duration, number of first-pass fixations, and average initial fixation position. GLMMs were used to analyze refixation probability. In all models, the visual segmentation cues were treated as a fixed factor, while participants and items were specified as crossed random effects, considering random intercepts and random slopes for both participants and items. When running the models, the maximum random effect structure model was first employed, and incrementally decremented until a good fit was achieved [57].

#### 2.2.1. Global Analysis

Descriptive statistics for each eye movement indicator in various visual segmentation cue conditions are presented in Table 1, and the statistical analysis results are presented in Table 2.

The results of the overall analysis of Experiment 1 showed that the average fixation duration and sentence reading time under the word spacing condition were shorter, with larger average saccade amplitude and fewer fixations and forward saccades, than the normal condition. This indicated that word spacing facilitated reading. Furthermore, the sentence reading times under the morpheme spacing and non-word spacing conditions were longer, with more fixations, forward saccades, and regressions, than the normal sentence condition. This suggested that morpheme spacing and non-word spacing hindered reading. Moreover, the morpheme spacing and non-word spacing conditions resulted in longer sentence reading times and more fixations, forward saccades, and regressions than the word spacing condition. This indicated that word spacing was more conducive to reading than morpheme spacing and non-word spacing. Overall, these results indicated that words were more likely than morphemes to be the basic processing unit in Tibetan reading.

In addition, Experiment 1 revealed that the average fixation duration was longer, and average saccade amplitude was smaller under the normal sentence and word spacing conditions than under the morpheme spacing condition. This could be because sentences in the normal sentence and word spacing conditions had a shorter physical length, higher information density in the horizontal spatial distribution, and larger amount of information in the same physical space than those in the morpheme spacing condition, leading to greater processing difficulty. Fixation duration reflects the difficulty of sentence processing [58]; greater sentence processing difficulty resulted in longer average fixation durations in the normal sentence and word spacing conditions, which showed smaller average saccade amplitudes. The number of artificially inserted spaces and sentence lengths in the word-spacing and non-word spacing conditions were consistent; however, average fixation duration was shorter, and average saccade amplitude was larger under the word spacing condition. This indicated that correct visual word segmentation cues facilitated word segmentation and reading, whereas incorrect visual word segmentation cues hindered it.

#### 2.2.2. Local Analysis

**Figure 3:**
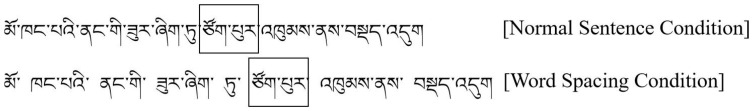
An illustration of local analysis based on areas of interest in different conditions (the boxes mean areas of interest). Translation: She is squatting in the corner of the room.

In Experiment 1, the interest areas of words under morpheme spacing and non-word spacing conditions differed in physical size and spatial distribution from those under normal sentence and word spacing conditions. The local analysis aimed to examine whether word spacing, compared to normal sentences, facilitated lexical recognition and saccade target selection. Therefore, based on previous studies [48], we only examined the local analysis indicators under normal sentence and word spacing conditions in the local analysis of Experiment 1. An area of interest consisting of a two-morpheme word composed of four horizontal letter spaces was selected from each experimental sentence (ensuring that the areas of interest under each condition were consistent in physical size, text content, and spatial distribution) to conduct a word-based local analysis of Tibetan sentences. These areas of interest did not appear at the beginning or end of the sentences to avoid interference from fixations related to the start and end of the reading [27,59]. Each condition contains 20 words in 20 sentences for comparing to their corresponding words from the other condition. The areas of interest for the local analysis are shown in Figure 3.

Descriptive statistics for each eye movement indicator under different visual segmentation cue conditions are presented in Table 3, and the statistical analysis results are shown in Table 4.

The results of the local analysis in Experiment 1 demonstrated that the word spacing condition had shorter gaze duration, total fixation duration, fewer first-pass fixations and lower total number of fixations than the normal sentence condition. The average initial fixation position was further from the word beginning and closer to the word center, with a lower refixation probability. This indicated that word spacing facilitated lexical recognition and aided in saccade target selection during reading.

Thus, Experiment 1 revealed that word spacing enhanced Tibetan reading and lexical recognition and facilitated saccade target selection. Words were more likely to be the basic processing unit in Tibetan reading and had a greater psychological reality than morphemes.

## 3. Experiment 2

This experiment employed eye-tracking technology and used Tibetan university students as participants. Four visual segmentation cues were set up (normal sentence, word boundary color alternation marking, morpheme boundary color alternation marking, and non-word color alternation marking) to investigate two questions, as follows: (1) whether color alternation markings can facilitate Tibetan reading and lexical recognition, and whether they are helpful for saccade target selection during the reading process; (2) whether the basic information processing unit in Tibetan reading is the morpheme or the word.

### 3.1. Materials and Methods

#### 3.1.1. Participants

Using the same standards and methods as in Experiment 1, we recruited 72 Tibetan university students (36 men and 36 women), none of whom had participated in Experiment 1, to participate in Experiment 2. The average age of the participants was 20.39 ± 1.36 years. The experiment was approved by the ethics committee of Tibet University. All participants provided written informed consent prior to participation. Participation was voluntary. The participants were compensated after completion of the experiment.

#### 3.1.2. Experimental Design

**Figure 4:**
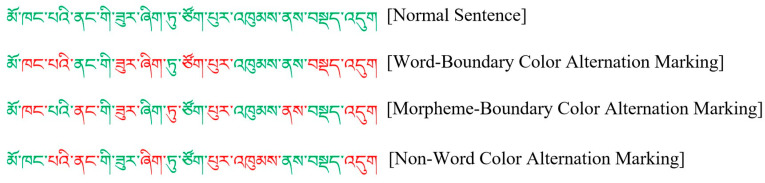
A sample of experimental materials. Translation: She is squatting in the corner of the room.

A single-factor within-subjects experimental design with four levels of visual segmentation cues (normal sentence, word boundary color alternation marking, morpheme boundary color alternation marking, and non-word color alternation marking) was employed. The word boundary color alternation marking condition referred to alternately marking adjacent words in Tibetan sentences in red and green. The morpheme boundary color alternation marking condition referred to alternately marking adjacent morphemes in Tibetan sentences in red and green. The non-word color alternation marking condition referred to the use of red and green colors alternating in Tibetan sentences to label elements that do not belong to independent word units. The number of color alternation markings under the word boundary color alternation marking condition was equal to that under the non-word color alternation marking condition, which provided a useful baseline for comparison with the word boundary color alternation marking condition [23]. The reason for using red and green color alternation markings is that people typically have good discrimination ability for red and green colors, even in the visual field far from the fovea [60]. Examples of the four conditions in Experiment 2 are illustrated in Figure 4.

#### 3.1.3. Experimental Materials

The materials used in Experiment 2 were the same as those in Experiment 1. The difference lay in the manipulation of visual segmentation cues. Experiment 1 manipulated spacing as the visual segmentation cue, whereas Experiment 2 employed red and green color alternation markings as the visual segmentation cue.

#### 3.1.4. Experimental Apparatus, Procedures, and Indicators

The experimental apparatus, procedures, and indicators were the same as those used in Experiment 1.

### 3.2. Data Analysis

The average accuracy of the participants’ responses was 89.85%, indicating that the participants read and understood the sentences carefully. The data exclusion criteria were the same as those used in Experiment 1. Consequently, 1.40% of the total data in the global analysis and 2.63% of the total data in the local analysis were excluded due to being invalid. The data analysis method was the same as that used in Experiment 1.

#### 3.2.1. Global Analysis

Descriptive statistics for each eye movement indicator under different visual segmentation cue conditions are presented in Table 5, and the statistical analysis results are presented in Table 6.

The global analysis results of Experiment 2 showed that the average fixation duration and sentence reading time under the word boundary color alternation marking condition were shorter, with fewer fixations and forward saccades, than under the normal sentence condition. This indicated that word boundary color alternation marking facilitated reading. Furthermore, the average fixation duration was longer (with word boundary color alternation marking condition *p* = 0.084); sentence reading time was longer; the average saccade amplitude was smaller; and more fixations, forward saccades, and regressions were present under the morpheme boundary color alternation marking condition and non-word color alternation marking condition than under the normal sentence condition. This suggested that morpheme boundary color alternation marking and non-word color alternation marking hindered reading. Moreover, the average fixation duration and sentence reading time were longer; the average saccade amplitude was smaller; and more fixations, forward saccades, and regressions occurred under the morpheme boundary color alternation marking condition and non-word color alternation marking condition than under the word boundary color alternation marking condition. This indicated that word boundary color alternation marking was more conducive to Tibetan reading than morpheme boundary color alternation marking and non-word color alternation marking. These results suggest that words are more likely than morphemes to be the basic processing units in Tibetan reading, supporting the findings of Experiment 1.

#### 3.2.2. Local Analysis

**Figure 5:**
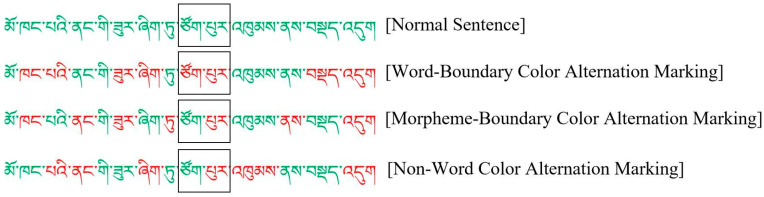
An illustration of local analysis based on areas of interest in different conditions (the boxes mean areas of interest). Translation: She is squatting in the corner of the room.

Experiment 2 employed color alternation markings as a visual segmentation cue to ensure that the spatial distribution, physical size, and text content of two-morpheme words (regions of interest) were consistent across the four conditions. Based on past studies, Experiment 2 analyzed the regions of interest under the four conditions to examine readers’ lexical recognition and saccade target selection across these conditions [23,61]. Similar to Experiment 1, a two-morpheme word composed of four horizontal letter spaces was selected as the region of interest in each experimental sentence, thereby ensuring consistency in physical size, text content, and spatial distribution across conditions. A word-based local analysis was conducted on Tibetan sentences, with regions of interest not appearing at the beginning or end of the sentences. The regions of interest for the local analysis are shown in Figure 5.

The descriptive statistics for each eye movement indicator under different visual segmentation cue conditions are presented in Table 7, and the statistical analysis results are shown in Table 8.

The local analysis results of Experiment 2 demonstrated that the gaze duration and total fixation duration were shorter, the number of first-pass fixations and total number of fixations were lower, and the refixation probability was smaller under the word boundary color alternation marking condition than under the normal sentence condition. This indicated that word boundary color alternation marking facilitated readers’ lexical recognition. However, no significant difference was found in the average initial fixation position between the word boundary color alternation marking condition and normal sentence condition. This suggested that word boundary color alternation marking, which is a visual word segmentation cue, did not affect readers’ saccade target selection. Furthermore, the non-word color alternation marking condition resulted in a longer gaze duration, total fixation duration, more first-pass fixations, higher total number of fixations, and a higher refixation probability than the normal sentence condition. The average initial fixation position was closer to the beginning of the word and further from the word center, although this result was marginally significant (*p* = 0.052). These results suggested that incorrect visual word segmentation cues were detrimental to readers’ word segmentation, which in turn hindered Tibetan lexical recognition and saccade target selection. Furthermore, the non-word color alternation marking condition exhibited a longer gaze duration and total fixation duration, more first-pass fixations, higher total number of fixations, and higher refixation probability than the word boundary color alternation marking condition. The initial fixation position was closer to the word beginning and further from the word center. The numbers of visual segmentation cues provided by word boundary color alternation marking and non-word color alternation marking were the same, indicating that words were important in Tibetan reading and that readers utilized the correct visual word segmentation cues to segment words for reading.

Thus, Experiment 2 found that word boundary color alternation marking promoted Tibetan reading and lexical recognition but did not affect saccade target selection. Words were more likely to be basic processing units in Tibetan reading and had a greater psychological reality than morphemes.

## 4. General Discussion

This study investigated whether visual word segmentation cues facilitate Tibetan reading, lexical recognition, and saccade target selection and whether the basic processing unit in Tibetan reading is the morpheme or the word. The results demonstrated that both interword spaces and color alternation markings facilitated reading and lexical recognition and that word spacing aided in the saccade target selection. Words were more likely to be basic processing units and had a greater psychological reality than morphemes.

### 4.1. The Effect of Interword Spaces in Tibetan Reading

Interword spaces facilitated Tibetan reading. This result differed from our experimental hypothesis and also differed from the results of previous studies, which found that interword spaces reduced reading speed in Thai and did not affect reading in Japanese and Chinese [16,17,18,19].

We propose two reasons for our finding. First, according to the hypothesis proposed by Bai et al. (2008), there was a trade-off between the interfering effect caused by readers’ familiarity with the text presentation and the promoting effect of visual word segmentation cues [18], and we speculated that Tibetan university students may not be highly familiar with the presentation of Tibetan text; therefore, the interword spaces are unlikely to have a significant impact on their familiarity with Tibetan texts. An important factor that may lead Tibetan college students to be unfamiliar with Tibetan texts could be that Tibetan university students experience language attrition. Language attrition is the reverse process of language acquisition, referring to the phenomenon in which language users experience a gradual decline in their ability to use the language over time due to a reduction or cessation in the use of a certain language [62,63]. Minority students living in China not only have to master their native languages but also learn Mandarin and other foreign languages, making language attrition a very common phenomenon [62]. In Tibet, Mandarin is used much more frequently than Tibetan, even in areas where Tibetan people live.

The objective reasons for native language attrition include the characteristics of the linguistic environment [62]. The participants in this study were Tibetan university students whose first language was Tibetan and second language was Chinese, respectively, making them Tibetan–Chinese bilinguals. If the native and second languages are from the same language family, the probability of native language attrition is greater. Based on the linguistic characteristics of Tibetan and Chinese, researchers believe that Tibetan and Chinese belonging to the same language family (the Sino-Tibetan language family), so they are more likely to be confused [62]. Confusion between the two languages is likely, leading to a high probability of language attrition among Tibetan–Chinese bilinguals. In addition, Tibetan university students are often immersed in a Chinese linguistic environment in their daily studies and life. Apart from students majoring in Tibetan, students in other majors rarely read books in the Tibetan language. Moreover, Tibetan people are divided into three major dialects: Weizang, Kang, and Amdo. Although all three dialects use the same script, their pronunciations differ vastly [64,65]. Therefore, many Tibetan university students from different dialectal backgrounds have to use Chinese for daily communication and reading.

These reasons may have led to a certain degree of language attrition among the participants, resulting in their insufficient experience in reading Tibetan and lower familiarity with the presentation of Tibetan texts. Consequently, the promoting effect of interword spaces on Tibetan reading outweighed the interfering effect caused by familiarity with the text presentation, leading to interword spaces facilitating Tibetan reading.

Second, the promoting effect of interword spaces on Tibetan reading may be due to the characteristics of the Tibetan text. Although Tibetan, Chinese, Japanese, and Thai texts do not have spaces as visual cues for word segmentation., Tibetan text is unique in that it contains an explicit visual word segmentation cue, known as tshegs, which is distinct from other languages. Existing research indicates that tshegs ensure the normal process of Tibetan reading, and that replacing tshegs with another visual word segmentation cue, such as a space, can facilitate Tibetan reading. This suggests that Tibetan university students rely on explicit visual segmentation cues (spaces and tshegs) to segment Tibetan texts [43]. Thus, Tibetan university students may be more accustomed to using explicit visual segmentation cues to segment text than Chinese, Japanese, and Thai students, leading to the artificial addition of interword spaces facilitating Tibetan reading.

Regarding the local analysis, indicators on the temporal dimension revealed that interword spaces facilitated lexical recognition of Tibetan reading. Specifically, gaze duration and total fixation time were shorter, and the number of first-pass fixations and total number of fixations were lower under the word spacing condition than used the normal sentence condition. This result is consistent with those of studies on Chinese and Thai reading [16,18,19], and supported the hypothesis that interword spaces facilitate lexical recognition.

In addition, the refixation probability was smaller, and the initial fixation position on words was closer to the word center under the word spacing condition than under the normal sentence condition. This indicated that the participants used word spacing for saccade target selection. Word spacing helped readers locate saccades in an optimal viewing position on a word, thereby facilitating lexical recognition. This result is consistent with the findings of Zang et al. (2013) and Ma et al. (2019) for Chinese reading [19,20]. This result also supported our hypothesis.

This result can be explained using the E-Z Reader model. The E-Z Reader model posits that readers can use low-spatial-frequency information during the pre-attentive visual processing stage (V stage) to guide saccade target selection [66,67], and that interword spaces provides low-spatial-frequency information that indicates word boundaries [66]. Interword spaces brings the letters that comprise words spatially closer, allowing readers to group spatially close letters into words through a parafoveal preview in the Tibetan text. This provides word boundary information, which helps readers locate their saccades at the optimal viewing position. The process by which readers group through a parafoveal preview may be based on the principle of proximity of the Gestalt laws of organization [68].

### 4.2. The Effect of Color Alternation Markings in Tibetan Reading

The interword spaces altered the spatial distribution of Tibetan sentences, elongated the physical length of the sentences, and reduced the processing efficiency of readers’ parafoveal vision. The insertion of spaces caused words near the fixation word to move away from the fixation, which may have resulted in words near the fixation falling into a visual area with lower visual acuity, thereby reducing the benefits of the parafoveal preview [21,22,23]. To eliminate the influence of this irrelevant factor on the results, Experiment 2 adopted the color alternation marking method to further investigate the effect of visual word segmentation cues in Tibetan reading.

The average fixation duration and sentence reading time were shorter, and fewer fixations and forward saccades were present under the word boundary color alternation marking condition than the normal sentence condition. This indicated that word boundary color alternation marking facilitated Tibetan reading. Furthermore, visual word segmentation cues promoted the reading of Tibetan sentences when controlling for their spatial distribution and physical length without changing the processing efficiency of the reader’s parafovea.

This result differs from our experimental hypothesis and diverges from the results of past studies. Previous studies have found that word boundary color alternation marking did not affect Chinese reading [23], whereas others have found that it impedes Chinese reading [19].

The reason for this may relate to the two speculations discussed above. First, Tibetan university students may have experienced language attrition, and their familiarity with the presentation of Tibetan texts may be low. This would lead to the promoting effect of word boundary color alternation marking on Tibetan reading being greater than the interfering effect caused by familiarity with text presentation, resulting in word boundary color alternation marking facilitating Tibetan reading. Second, Tibetan university students may be more accustomed to using low-level visual segmentation cues for word segmentation during Tibetan reading than Chinese readers, thereby aiding in Tibetan reading.

Word boundary color alternation marking facilitated lexical recognition in Tibetan reading. This result is consistent with the findings of Pan et al. (2021) for Chinese reading and those of Zhou et al. (2018) among native speakers of Chinese [23,24]. This supported our hypothesis that color alternation markings facilitate local lexical recognition. However, the results differed from those of Ma et al. (2019), who found that color alternation markings did not facilitate Chinese lexical recognition [19]. Nevertheless, as the global analysis of Experiment 2 showed that word boundary color alternation marking facilitated Tibetan reading, the finding in the local analysis that color alternation markings promoted lexical recognition is reasonable, indicating that color alternation markings facilitated Tibetan reading by promoting local lexical recognition.

Furthermore, color alternation markings reduced the probability of readers’ re-gazing. This was consistent with the results of Zhou et al. (2018) for Chinese reading [23]. However, word boundary color alternation marking did not affect readers’ saccade target selection. This was consistent with the results of Ma et al. (2019) for Chinese reading but inconsistent with those of Zhou et al. (2018) and with our hypothesis [19,23]. This could be because Tibetan readers tended to use the principle of proximity of the Gestalt laws of organization when grouping adjacent letters into words through a parafoveal preview rather than the principle of similarity (letters of the same color making readers perceive them as a whole) [68,69].

### 4.3. The Basic Information Processing Units in Tibetan Reading

Tibetan texts contain tshegs. Wang et al. (2023) found that removing tshegs decreased the efficiency of Tibetan reading. This indicated that tshegs are an effective visual word segmentation cue that ensures the normal progression of Tibetan reading [43]. Thus, Tibetan university students may be accustomed to relying on the visual word segmentation cue of tshegs for reading morpheme by morpheme, and morphemes may be the basic information processing unit in Tibetan reading.

Moreover, the attributes of words in Tibetan reading affected readers’ fixation duration. For instance, Tibetan readers spent significantly less time fixating on high- than low-frequency words [44,45]. Thus, words may be the basic information processing unit in Tibetan reading.

Therefore, the word spacing condition had fewer fixations, forward saccades, and shorter sentence reading time than the normal sentence condition. The word boundary color alternation marking condition had fewer fixations, forward saccades, and shorter sentence reading time than the normal sentence condition. The morpheme spacing condition had more fixations, forward saccades, regressions, and longer sentence reading time than the normal sentence condition. The morpheme color alternation marking condition had more fixations, forward saccades, regressions, and longer sentence reading time than the normal sentence condition. The word spacing condition had fewer fixations, forward saccades, regressions, and shorter sentence reading time than the morpheme spacing condition. The word boundary color alternation marking condition had fewer fixations, forward saccades, regressions, and shorter sentence reading time than the morpheme color alternation marking condition.

Thus, words were more likely than morphemes to be the basic unit of information processing in Tibetan reading. This result was in line with those of studies that considered words to be the basic unit of information processing in Chinese reading [31,32,33,34,35]. However, the basic unit of information processing in Tibetan reading may also be influenced by various factors, which will be discussed in detail in the Limitations and Prospects section.

This study sheds light on the word segmentation mechanism in Tibetan reading, reveals the nature and rules of Tibetan reading activities from the perspectives of reading psychology and cognitive psychology, and provides basic data for the improvement of eye movement control models in alphabetic writing systems. Moreover, the findings provide practical guidance for exploring ways to improve the efficiency of Tibetan reading and a scientific basis for word segmentation in Tibetan information processing and modern Tibetan intelligent systems.

### 4.4. Limitations and Prospects

Yan et al. (2013) investigated the impact of interword spaces in Chinese texts on the reading of second-grade elementary students and found no significant difference in sentence reading time when second-grade students read normal sentences and those under character spacing and word spacing conditions [70]. This result suggests that both characters and words may be the basic units of information processing in Chinese reading. Therefore, future research should investigate the basic units of information processing in Tibetan reading among elementary school students.

This study examined only four conditions, including normal sentences, word spacing, morpheme spacing, and non-word spacing. Words were more likely than morphemes to be the basic unit of information processing in Tibetan reading. Conversely, some studies suggest that chunks, psycholinguistic words, and prosodic words may be the basic units of information processing in Chinese reading [71,72,73,74,75]. Whether these conclusions are also applicable to Tibetan has not yet been studied. Therefore, future research could set chunks, psycholinguistic words, and prosodic words as experimental conditions based on the original experimental conditions to further investigate the basic units of information processing in Tibetan reading.

This study found that adding spaces as low-level visual word segmentation cues to Tibetan texts helped readers segment words, thereby promoting reading. However, although Tibetan text does not have spaces as inherent low-level visual word segmentation cues, reading proceeds normally. Therefore, we speculate that Tibetan readers may use high-level linguistic word segmentation cues to segment words, thereby ensuring normal reading progress. Studies have found that character positional frequency information and word formation probabilities in Chinese reading are effective linguistic word segmentation cues [76,77]. Furthermore, character positional frequency information is an effective linguistic word segmentation cue in Thai reading [78]. Therefore, future research should examine the effect of high-level linguistic word segmentation cues in Tibetan reading based on comprehensive corpus data, thereby revealing the word segmentation mechanism in Tibetan reading.

## 5. Conclusions

Artificially incorporated interword spaces facilitate Tibetan reading, lexical recognition, and saccade target selection. Color alternation markings facilitate Tibetan reading and lexical recognition but do not affect saccade target selection. Words are more likely to be the basic unit of information processing in Tibetan reading and have a greater psychological reality than morphemes.

## Figures and Tables

**Table 1:** Means and standard deviations of eye movement indicators in the global analysis.

Visual Segmentation Cues	Average Fixation Duration (ms)	Average Saccade Amplitude (Horizontal Letters)	Number of Fixations	Sentence Reading Time (ms)	Number of Forward Saccades	Number of Regressions
Normal Sentence	242 (26)	1.80 (0.30)	16.51 (3.56)	6019 (1402)	11.75 (2.16)	3.18 (1.04)
Word Spacing	232 (24)	2.04 (0.36)	15.50 (3.04)	5566 (1156)	11.22 (1.92)	3.13 (1.17)
Morpheme Spacing	225 (25)	2.41 (0.47)	19.54 (4.91)	6778 (1819)	14.40 (3.38)	4.04 (1.85)
Non-Word Spacing	243 (26)	1.94 (0.32)	20.83 (5.79)	7552 (2210)	14.55 (3.77)	4.34 (1.88)

(standard deviations in parentheses).

**Table 2:** Statistical analysis of eye movement indicators in the global analysis.

		Average Fixation Duration (ms)	Average Saccade Amplitude (Horizontal Letters)	Number of Fixations	Sentence Reading Time (ms)	Number of Forward Saccades	Number of Regressions
Normal Sentence/Word Spacing	*b*	0.044	−0.127	0.063	0.083	0.044	0.03
	*SE*	0.007	0.013	0.015	0.016	0.011	0.03
	*t*	6.507 ***	−9.993 ***	4.273 ***	5.145 ***	4.074 ***	0.99
Normal Sentence/Morpheme Spacing	*b*	0.075	−0.294	−0.151	−0.116	−0.196	−0.201
	*SE*	0.007	0.014	0.019	0.022	0.02	0.037
	*t*	10.022 ***	−21.353 ***	−7.877 ***	−5.209 ***	−9.705 ***	−5.406 ***
Normal Sentence/Non-Word Spacing	*b*	−0.005	−0.074	−0.218	−0.227	−0.199	−0.282
	*SE*	0.006	0.013	0.022	0.022	0.019	0.038
	*t*	−0.843	−5.838 ***	−10.072 ***	−10.344 ***	−10.427 ***	−7.495 ***
Word Spacing/Morpheme Spacing	*b*	0.032	−0.167	−0.214	−0.199	−0.24	−0.23
	*SE*	0.008	0.013	0.022	0.024	0.022	0.037
	*t*	3.891 ***	−13.349 ***	−9.863 ***	−8.391 ***	−11.118 ***	−6.205 ***
Word Spacing/Non-Word Spacing	*b*	−0.048	0.054	−0.281	−0.31	−0.244	−0.311
	*SE*	0.008	0.014	0.025	0.026	0.022	0.035
	*t*	−5.751 ***	3.785 ***	−11.393 ***	−12.073 ***	−11.263 ***	−8.924 ***

The *SE* refers to *b*; *** *p* < 0.001.

**Table 3:** Means and standard deviations of eye movement indicators in the local analysis.

Visual Segmentation Cues	First Fixation Duration (ms)	Gaze Duration (ms)	Number of First-Pass Fixations	Total Fixation Duration (ms)	Total Number of Fixations	Average Initial Fixation Position (Horizontal Letters)	Refixation Probability (%)
Normal Sentence	248 (36)	407 (96)	1.64 (0.31)	544 (147)	2.21 (0.52)	1.43 (0.30)	0.50 (0.17)
Word Spacing	247 (38)	356 (84)	1.48 (0.32)	466 (124)	1.97 (0.50)	1.69 (0.36)	0.38 (0.20)

(standard deviations in parentheses).

**Table 4:** Statistical analysis results of normal and interword eye movement indicators in the local analysis.

		First Fixation Duration (ms)	Gaze Duration (ms)	Number of First-Pass Fixations	Total Fixation Duration (ms)	Total Number of Fixations	Average Initial Fixation Position (Horizontal Letters)	Refixation Probability (%)
Normal Sentence/Word Spacing	*b*	0.009	0.125	0.103	0.15	0.119	−0.27	0.58
	*SE*	0.013	0.023	0.019	0.023	0.022	0.045	0.081
	*t/z*	0.651	5.472 ***	5.398 ***	6.540 ***	5.357 ***	−5.954 ***	7.130 ***

The *SE* refers to *b*; *** *p* < 0.001.

**Table 5:** Means and standard deviations of eye movement indicators in the global analysis.

Visual Segmentation Cues	Average Fixation Duration (ms)	Average Saccade Amplitude (Horizontal Letters)	Number of Fixations	Sentence Reading Time (ms)	Number of Forward Saccades	Number of Regressions
Normal Sentence	239 (25)	1.95 (0.36)	16.28 (3.97)	6016 (1407)	11.37 (2.62)	3.40 (1.25)
Word Boundary Color Alternation Marking	236 (25)	1.96 (0.32)	15.73 (3.51)	5737 (1151)	10.90 (2.35)	3.37 (1.19)
Morpheme Boundary Color Alternation Marking	241 (28)	1.89 (0.34)	17.63 (4.98)	6504 (1602)	12.23 (3.44)	3.76 (1.57)
Non-Word Color Alternation Marking	242 (27)	1.84 (0.33)	18.51 (4.59)	6774 (1479)	12.56 (2.87)	4.00 (1.53)

(standard deviations in parentheses).

**Table 6:** Statistical analysis of eye movement indicators in the overall analysis.

		Average Fixation Duration (ms)	Average Saccade Amplitude (Horizontal Letters)	Number of Fixations	Sentence Reading Time (ms)	Number of Forward Saccades	Number of Regressions
Normal Sentence/Word Boundary	*b*	0.012	−0.008	0.029	0.042	0.038	0.02
	*SE*	0.005	0.011	0.013	0.015	0.011	0.024
	*t*	2.429 *	−0.706	2.193 *	2.873 **	3.390 **	0.854
Normal Sentence/Morpheme Boundary	*b*	−0.01	0.027	−0.073	−0.079	−0.067	−0.077
	*SE*	0.006	0.01	0.017	0.02	0.016	0.021
	*t*	−1.753 ^§^	2.614 *	−4.313 ***	−4.044 ***	−4.292 ***	−3.673 ***
Normal Sentence/Non-Word	*b*	−0.011	0.055	−0.127	−0.131	−0.099	−0.142
	*SE*	0.006	0.009	0.013	0.015	0.011	0.021
	*t*	−2.012 *	5.946 ***	−10.062 ***	−8.499 ***	−9.264 ***	−6.796 ***
Word Boundary/Morpheme Boundary	*b*	−0.022	0.034	−0.102	−0.121	−0.105	−0.099
	*SE*	0.005	0.01	0.015	0.016	0.014	0.026
	*t*	−4.387 ***	3.300 **	−6.789 ***	−7.577 ***	−7.625 ***	−3.827 ***
Word Boundary/Non-Word	*b*	−0.024	0.063	−0.156	−0.173	−0.137	−0.162
	*SE*	0.006	0.012	0.012	0.015	0.011	0.022
	*t*	−4.254 ***	5.453 ***	−13.234 ***	−11.857 ***	−12.421 ***	−7.387 ***

The *SE* refers to *b*; * *p* < 0.05; ** *p* < 0.01; *** *p* < 0.001; ^§^ *p* < 0.1.

**Table 7:** Means and standard deviations of eye movement indicators in the local analysis.

Visual Segmentation Cues	First Fixation Duration (ms)	Gaze Duration (ms)	Number of First-Pass Fixations	Total Fixation Duration (ms)	Total Number of Fixations	Average Initial Fixation Position (Horizontal Letters)	Refixation Probability (%)
Normal Sentence	247 (32)	394 (88)	1.62 (0.33)	543 (142)	2.28 (0.64)	1.48 (0.37)	0.49 (0.20)
Word Boundary Color Alternation Marking	247 (38)	382 (91)	1.57 (0.33)	516 (135)	2.17 (0.63)	1.49 (0.35)	0.45 (0.22)
Morpheme Boundary Color Alternation Marking	249 (39)	406 (96)	1.65 (0.32)	586 (198)	2.43 (0.83)	1.44 (0.40)	0.51 (0.21)
Non-Word Color Alternation Marking	249 (36)	413 (89)	1.69 (0.35)	605 (179)	2.52 (0.81)	1.42 (0.32)	0.52 (0.19)

(standard deviations in parentheses).

**Table 8:** Statistical analysis results of eye movement indicators in the local analysis.

		First Fixation Duration (ms)	Gaze Duration (ms)	Number of First-Pass Fixations	Total Fixation Duration (ms)	Total Number of Fixations	Average Initial Fixation Position (Horizontal Letters)	Refixation Probability (%)
Normal Sentence/Word Boundary	*b*	0.001	0.038	0.03	0.054	0.044	−0.006	0.168
	*SE*	0.013	0.018	0.015	0.022	0.017	0.038	0.081
	*t/z*	0.1	2.100 *	2.070 *	2.519 *	2.548 *	−0.167	2.075 *
Normal Sentence/Morpheme Boundary	*b*	−0.011	−0.024	−0.021	−0.061	−0.05	0.039	−0.133
	*SE*	0.013	0.018	0.015	0.029	0.017	0.038	0.081
	*t/z*	−0.871	−1.305	−1.429	−2.126 *	−2.894 **	1.032	−1.64
Normal Sentence/Non-Word	*b*	−0.006	−0.042	−0.033	−0.101	−0.093	0.074	−0.161
	*SE*	0.013	0.018	0.015	0.022	0.017	0.038	0.081
	*t/z*	−0.491	−2.317 *	−2.227 *	−4.552 ***	−5.338 ***	1.945 ^§^	−1.995 *
Word Boundary/Morpheme Boundary	*b*	−0.012	−0.061	−0.051	−0.115	−0.095	0.046	−0.301
	*SE*	0.013	0.018	0.015	0.022	0.017	0.038	0.081
	*t/z*	−0.972	−3.407 ***	−3.500 ***	−5.147 ***	−5.442 ***	1.2	−3.708 ***
Word Boundary/Non-Word	*b*	−0.007	−0.079	−0.063	−0.155	−0.137	0.08	−0.329
	*SE*	0.013	0.018	0.015	0.022	0.017	0.038	0.081
	*t/z*	−0.592	−4.426 ***	−4.302 ***	−6.948 ***	−7.891 ***	2.113 *	−4.060 ***

The *SE* refers to *b*; * *p* < 0.05; ** *p* < 0.01; *** *p* < 0.001; ^§^ *p* < 0.1.

## Data Availability

The original contributions presented in the study are included in the Supplementary Material; further inquiries can be directed to the corresponding authors.

## Supplementary Materials

The following supporting information can be downloaded at: https://www.mdpi.com/article/10.3390/brainsci14100964/s1, File S1: Supplementary Materials of The Effect of Visual Word Segmentation Cuesin Tibetan Reading.
