# Supplementary material for: The Effect of Visual Word Segmentation Cues in Tibetan Reading
Source: Brain Sci. 2024 Sep 25;14(10):964. doi: 10.3390/brainsci14100964 (PMC11505889; doi:10.3390/brainsci14100964)
Supplement: Supplementary file 1 [file brainsci-14-00964-s001.zip › Data Analysis.pdf]

Global Analysis In Experiment 1

The analysis of average fixation duration revealed that the duration in the normal sentence condition was significantly greater than that observed in both the interword spacing condition ($b = 0.044$, $SE = 0.007$, $t = 6.507$, $p < 0.001$) and the intracharacter spacing condition ($b = 0.075$, $SE = 0.007$, $t = 10.022$, $p < 0.001$). No significant difference was found in average fixation duration between the normal sentence condition and the nonword spacing condition ($b = -0.005$, $SE = 0.006$, $t = -0.843$, $p = 0.402$). Furthermore, the average fixation duration in the interword spacing condition was significantly longer than that in the intracharacter spacing condition ($b = 0.032$, $SE = 0.008$, $t = 3.981$, $p < 0.001$). Additionally, the nonword spacing condition exhibited a significantly longer average fixation duration compared to the intracharacter spacing condition ($b = 0.080$, $SE = 0.007$, $t = 11.890$, $p < 0.001$), and it was also significantly longer than the interword spacing condition ($b = 0.048$, $SE = 0.008$, $t = 5.751$, $p < 0.001$).

They analysis of average saccade amplitude revealed that the amplitudes recorded under the interword spacing condition ($b = 0.127$, $SE = 0.013$, $t = 9.993$, $p < 0.001$), intracharacter spacing condition ($b = 0.294$, $SE = 0.014$, $t = 21.353$, $p < 0.001$), and nonword spacing condition ($b = 0.074$, $SE = 0.013$, $t = 5.838$, $p < 0.001$) were significantly greater than those observed in the normal sentence condition. Furthermore, the average saccade amplitude in the interword spacing condition was significantly less than that in the

intracharacter spacing condition (b = -0.167, SE = 0.013, t = -13.349, p < 0.001). In contrast, the average saccade amplitude in the interword spacing condition was significantly longer than that in the nonword spacing condition (b = 0.054, SE = 0.014, t = 3.785, p < 0.001). Additionally, the average saccade amplitude in the nonword spacing condition was significantly lower than that under the intracharacter spacing condition (b = -0.221, SE = 0.013, t = -16.684, p < 0.001).

The analysis of fixation counts revealed that the the number of fixations in the interword spacing condition was significantly lower compared to the normal sentence condition (b = -0.063, SE = 0.015, t = -4.273, p < 0.001), the intracharacter spacing condition (b = -0.214, SE = 0.022, t = -9.863, p < 0.001), and the nonword spacing condition (b = -0.281, SE = 0.025, t = -11.393, p < 0.001). Furthermore, the number of fixations in the normal sentence condition was significantly lower than that under the intracharacter spacing condition (b = -0.151, SE = 0.019, t = -7.877, p < 0.001) and the nonword spacing condition (b = -0.218, SE = 0.022, t = -10.072, p < 0.001). Additionally, the number of fixations under the intracharacter spacing condition was significantly lower than that in the nonword spacing condition (b = -0.068, SE = 0.015, t = -4.610, p < 0.001).

In terms of sentence reading time, the interword spacing condition exhibited significantly shorter reading times than the normal sentence condition (b = -0.083, SE = 0.016, t = -5.145, p < 0.001), the intracharacter spacing condition (b = -0.199, SE = 0.024, t = -8.391, p < 0.001), and the nonword spacing condition (b

= -0.310, $SE = 0.026$, $t = -12.073$, $p < 0.001$). The reading time for the normal sentence condition was also significantly shorter than that for the intracharacter spacing condition ($b = -0.116$, $SE = 0.022$, $t = -5.209$, $p < 0.001$) and the nonword spacing condition ($b = -0.227$, $SE = 0.022$, $t = -10.344$, $p < 0.001$). Lastly, the reading time in the intracharacter spacing condition was significantly shorter than that under the nonword spacing condition ($b = -0.111$, $SE = 0.016$, $t = -6.746$, $p < 0.001$).

The analysis of forward saccades, revealed that the count of forward saccades under the interword spacing condition was significantly lower than that under the normal sentence condition ($b = -0.044$, $SE = 0.011$, $t = -4.074$, $p < 0.001$), the intracharacter spacing condition ($b = -0.240$, $SE = 0.022$, $t = -11.118$, $p < 0.001$), and the nonword spacing condition ($b = -0.244$, $SE = 0.022$, $t = -11.263$, $p < 0.001$). Furthermore, the number of forward saccades in the normal sentence condition was significantly less than that observed in the intracharacter spacing condition ($b = -0.196$, $SE = 0.020$, $t = -9.705$, $p < 0.001$) and the nonword condition ($b = -0.199$, $SE = 0.019$, $t = -10.427$, $p < 0.001$). However, no significant difference was found in the number of forward saccades between the intracharacter spacing condition and the nonword spacing condition ($b = -0.004$, $SE = 0.013$, $t = -0.310$, $p = 0.758$).

In terms of regressions, the comparison between the interword spacing condition and the normal sentence condition did not yield a significant difference ($b = -0.030$, $SE = 0.030$, $t = -0.990$, $p = 0.326$). Conversely, the number

of regressions in the interword spacing condition was significantly lower than in the intracharacter spacing condition (b = -0.230, SE = 0.037, t = -6.205, p < 0.001) and the nonword spacing condition (b = -0.311, SE = 0.035, t = -8.924, p < 0.001). Additionally, the normal sentence condition exhibited significantly fewer regressions compared to the intracharacter spacing condition (b = -0.201, SE = 0.037, t = -5.406, p < 0.001) and the nonword spacing condition (b = -0.282, SE = 0.038, t = -7.495, p < 0.001). Lastly, the intracharacter spacing condition showed a significantly lower number of regressions than the nonword spacing condition (b = -0.081, SE = 0.025, t = -3.185, p = 0.002).

Local Analysis In Experiment 1

In terms of first fixation duration, no statistically significant difference was observed between the normal sentence condition and the interword spacing condition (b = 0.009, SE = 0.013, t = 0.651, p = 0.517).

Regarding gaze duration, it was found that the gaze duration in the interword spacing condition was significantly reduced compared to that in the normal sentence condition (b = -0.125, SE = 0.023, t = -5.472, p < 0.001).

When examining the number of first-pass fixations, the interword spacing condition resulted in a significantly lower number of first-pass fixations than the normal sentence condition (b = -0.103, SE = 0.019, t = -5.398, p < 0.001).

For total fixation duration, participants exhibited significantly shorter total fixation durations in the interword spacing condition compared to the normal sentence condition (b = -0.150, SE = 0.023, t = -6.540, p < 0.001).

In terms of the total number of fixations, the interword spacing condition also led to a significantly reduced total number of fixations compared to the normal sentence condition (b = -0.119, SE = 0.022, t = -5.357, p < 0.001).

Concerning the average initial fixation position, the interword spacing condition resulted in an average initial fixation position that was further from the beginning of the word and closer to the center of the word when compared to the normal sentence condition (b = 0.270, SE = 0.045, t = 5.954, p < 0.001).

Lastly, the probability of refixation was significantly lower in the interword spacing condition than in the normal sentence condition (b = -0.580, SE = 0.081, z = -7.130, p < 0.001).

Global Analysis In Experiment 2

In terms of average fixation duration, the results indicated that the fixation duration in the interword color alternation condition was significantly shorter compared to the normal sentence condition (b = -0.012, SE = 0.005, t = -2.429, p = 0.018), the intracharacter color alternation condition (b = -0.022, SE = 0.005, t = -4.387, p < 0.001), and the nonword color alternation condition (b = -0.024, SE = 0.006, t = -4.254, p < 0.001). Furthermore, the average fixation duration under the normal sentence condition was marginally significantly shorter than that under the intracharacter color alternation condition (b = -0.010, SE = 0.006, t = -1.753, p = 0.084) and significantly shorter than in the nonword color

alternation condition (*b* = -0.011, *SE* = 0.006, *t* = -2.012, *p* = 0.048). No significant difference was observed in average fixation duration between the intracharacter color alternation condition and the nonword color alternation condition (*b* = -0.002, *SE* = 0.007, *t* = -0.251, *p* = 0.802).

Regarding average saccade amplitude, the analysis revealed no significant difference between the interword color alternation condition and the normal sentence condition (*b* = 0.008, *SE* = 0.011, *t* = 0.706, *p* = 0.483). However, the average saccade amplitude under the interword color alternation condition was significantly greater than that in the intracharacter color alternation condition (*b* = 0.034, *SE* = 0.010, *t* = 3.300, *p* = 0.002) and the nonword color alternation condition (*b* = 0.063, *SE* = 0.012, *t* = 5.453, *p* < 0.001). Additionally, the average saccade amplitude in the normal sentence condition was significantly longer than that in both the intracharacter color alternation condition (*b* = 0.027, *SE* = 0.010, *t* = 2.614, *p* = 0.011) and the nonword color alternation condition (*b* = 0.055, *SE* = 0.009, *t* = 5.946, *p* < 0.001). The average saccade amplitude in the intracharacter color alternation condition was significantly longer than that under the nonword color alternation condition (*b* = 0.029, *SE* = 0.011, *t* = 2.731, *p* = 0.008).

The analysis of fixation counts revealed that the number of fixations in the interword color alternation condition was significantly lower than in the normal sentence condition (*b* = -0.029, *SE* = 0.013, *t* = -2.193, *p* = 0.032), the

intracharacter color alternation condition (b = -0.102, SE = 0.015, t = -6.789, $p <$ 0.001), and the nonword color alternation condition (b = -0.156, SE = 0.012, t = -13.234, $p < 0.001$). Furthermore, the number of fixations in the normal sentence condition was significantly lower than that in the intracharacter color alternation condition (b = -0.073, SE = 0.017, t = -4.313, $p < 0.001$) and the nonword color alternation condition (b = -0.127, SE = 0.013, t = -10.062, $p <$ 0.001). Additionally, the number of fixations under the intracharacter color alternation condition was significantly fewer than that under the nonword color alternation condition (b = -0.055, SE = 0.015, t = -3.696, $p < 0.001$).

In terms of sentence reading time, the interword color alternation condition exhibited significantly shorter reading times comapred to the normal sentence condition (b = -0.042, SE = 0.015, t = -2.873, $p = 0.005$), the intracharacter color alternation condition (b = -0.121, SE = 0.016, t = -7.577, $p < 0.001$), and the nonword color alternation condition (b = -0.173, SE = 0.015, t = -11.857, $p <$ 0.001). The reading time for the normal sentence condition was significantly shorter than that for the intracharacter color alternation condition (b = -0.079, SE = 0.020, t = -4.044, $p < 0.001$) and the nonword color alternation condition (b = -0.131, SE = 0.015, t = -8.499, $p < 0.001$). The reading time in the intracharacter color alternation condition was significantly shorter than in the nonword color alternation condition (b = -0.052, SE = 0.019, t = -2.789, $p = 0.007$).

The analysis of forward saccades revealed that the count of forward saccades in the interword color alternation condition was significantly lower than in

the normal sentence condition (b = -0.038, SE = 0.011, t = -3.390, p = 0.001), the intracharacter color alternation condition (b = -0.105, SE = 0.014, t = -7.625, $p <$ 0.001), and the nonword color alternation condition (b = -0.137, SE = 0.011, t = -12.421, $p <$ 0.001).Furthermore, the number of forward saccades in the normal sentence condition was significantly less than that observed in the intracharacter color alternation condition (b = -0.067, SE = 0.016, t = -4.292, $p <$ 0.001) and the nonword color alternation condition (b = -0.099, SE = 0.011, t = -9.264, $p <$ 0.001). Additionally, the forward saccades recorded in the intracharacter color alternation condition were significantly fewer than those in the nonword color alternation condition (b = -0.033, SE = 0.013, t = -2.486, p = 0.015).

In terms of regressions, no significant difference was found between the interword color alternation condition and the normal sentence condition (b = -0.020, SE = 0.024, t = -0.854, p = 0.396). However, the number of regressions in the interword color alternation condition was significantly lower than in the intracharacter color alternation condition (b = -0.099, SE = 0.026, t = -3.827, $p <$ 0.001) and the nonword color alternation condition (b = -0.162, SE = 0.022, t = -7.387, $p <$ 0.001). The regressions in the normal sentence condition were also significantly fewer than those in the intracharacter color alternation condition (b = -0.077, SE = 0.021, t = -3.673, $p <$ 0.001) and the nonword color alternation condition (b = -0.142, SE = 0.021, t = -6.796, $p <$ 0.001). The number of regressions in the intracharacter color alternation condition was significantly

lower than that in the nonword color alternation condition (b = -0.063, SE = 0.029, t = -2.161, p = 0.034).

Local Analysis In Experiment 2

The analysis of first fixation duration revealed no statistically significant differences between the interword color alternation condition and the normal sentence condition (b = -0.001, SE = 0.013, t = -0.100, p = 0.920). Additionally, comparisons between the interword color alternation condition and both the intracharacter color alternation condition (b = -0.012, SE = 0.013, t = -0.972, p = 0.331) and the nonword color alternation condition (b = -0.007, SE = 0.013, t = -0.592, p = 0.554) also showed no significant differences. Similarly, no significant differences were found between the normal sentence condition and the intracharacter color alternation condition (b = -0.011, SE = 0.013, t = -0.871, p = 0.384), nor between the normal sentence condition and the nonword color alternation condition (b = -0.006, SE = 0.013, t = -0.491, p = 0.624).

In terms of gaze duration, the interword color alternation condition resulted in significantly shorter gaze durations compared to the normal sentence condition (b = -0.038, SE = 0.018, t = -2.100, p = 0.036), the intracharacter color alternation condition (b = -0.061, SE = 0.018, t = -3.407, $p < 0.001$), and the nonword color alternation condition (b = -0.079, SE = 0.018, t = -4.426, $p < 0.001$). However, no significant difference in gaze duration was observed between the normal sentence condition and the intracharacter color alternation condition (b = -0.024, SE = 0.018, t = -1.305, p = 0.192). Conversely,

the gaze duration in the normal sentence condition was significantly shorter than that in the nonword color alternation condition (b = -0.042, SE = 0.018, t = -2.317, p = 0.021).

The analysis of first-pass fixations revealed that the number of fixations under the interword color alternation condition was significantly lower than in the normal sentence condition (b = -0.030, SE = 0.015, t = -2.070, p = 0.039), the intracharacter color alternation condition (b = -0.051, SE = 0.015, t = -3.500, p < 0.001), and the nonword color alternation condition (b = -0.063, SE = 0.015, t = -4.302, p < 0.001). However, no significant difference was observed in the number of first-pass fixations between the normal sentence condition and the intracharacter color alternation condition (b = -0.021, SE = 0.015, t = -1.429, p = 0.153). In contrast, the normal sentence condition exhibited significantly fewer first-pass fixations compared to the nonword color alternation condition (b = -0.033, SE = 0.015, t = -2.227, p = 0.026).

Regarding total fixation duration, the interword color alternation condition demonstrated a significantly shorter total fixation duration than the normal sentence condition (b = -0.054, SE = 0.022, t = -2.519, p = 0.013), the intracharacter color alternation condition (b = -0.115, SE = 0.022, t = -5.147, p < 0.001), and the nonword color alternation condition (b = -0.155, SE = 0.022, t = -6.948, p < 0.001). Additionally, the total fixation duration in the normal sentence condition was significantly shorter than that in the intracharacter color alternation condition (b = -0.061, SE = 0.029, t = -2.126, p = 0.037) and the

nonword color alternation condition (b = -0.101, SE = 0.022, t = -4.552, $p <$ 0.001).

The analysis of total fixations revealed that the number of fixations observed in the interword color alternation condition was significantly lower than that recorded in the normal sentence condition (b = -0.044, SE = 0.017, t = -2.548, p = 0.011), the intracharacter color alternation condition (b = -0.095, SE = 0.017, t = -5.442, $p < 0.001$), and the nonword color alternation condition (b = -0.137, SE = 0.017, t = -7.891, $p < 0.001$). Furthermore, the total fixations in the normal sentence condition were significantly fewer than those in the intracharacter color alternation condition (b = -0.050, SE = 0.017, t = -2.894, p = 0.004) and the nonword color alternation condition (b = -0.093, SE = 0.017, t = -5.338, $p <$ 0.001).

The analysis of average initial fixation position revealed no statistically significant differences between the interword color alternation condition and the normal sentence condition (b = 0.006, SE = 0.038, t = 0.167, p = 0.867). Similarly, no significant difference was observed when comparing the interword color alternation condition with the intracharacter color alternation condition (b = 0.046, SE = 0.038, t = 1.200, p = 0.230). In contrast, the average initial fixation position in the interword color alternation condition was found to be further from the beginning of the word and closer to the center when compared to the nonword color alternation condition (b = 0.080, SE = 0.038, t = 2.113, p = 0.035). Likewise, the average initial fixation position in the normal

sentence condition also exhibited a tendency to be further from the beginning and closer to the center of the word compared to the nonword color alternation condition (*b* = 0.074, *SE* = 0.038, *t* = 1.945, *p* = 0.052), although this finding approached significance. No significant difference was identified in the average initial fixation position between the normal sentence condition and the intracharacter color alternation condition (*b* = 0.039, *SE* = 0.038, *t* = 1.032, *p* = 0.302).

Reading refixation probability, the interword color alternation condition demonstrated a significantly lower refixation probability compared to the normal sentence condition (*b* = -0.168, *SE* = 0.081, *z* = -2.075, *p* = 0.038), the intracharacter color alternation condition (*b* = -0.301, *SE* = 0.081, *z* = -3.708, $p < 0.001$), and the nonword color alternation condition (*b* = -0.329, *SE* = 0.081, *z* = -4.060, $p < 0.001$). No significant difference in refixation probability was found between the normal sentence condition and the intracharacter color alternation condition (*b* = -0.133, *SE* = 0.081, *z* = -1.640, *p* = 0.101); however, the refixation probability in the normal sentence condition was significantly lower than that in the nonword color alternation condition (*b* = -0.161, *SE* = 0.081, *z* = -1.995, *p* = 0.046).
